# Supplementary figures and images for: Modeling the role of the thalamus in resting-state functional connectivity: Nature or structure
Source: PLoS Comput Biol. 2023 Aug 3;19(8):e1011007. doi: 10.1371/journal.pcbi.1011007 (PMC10426958; doi:10.1371/journal.pcbi.1011007)

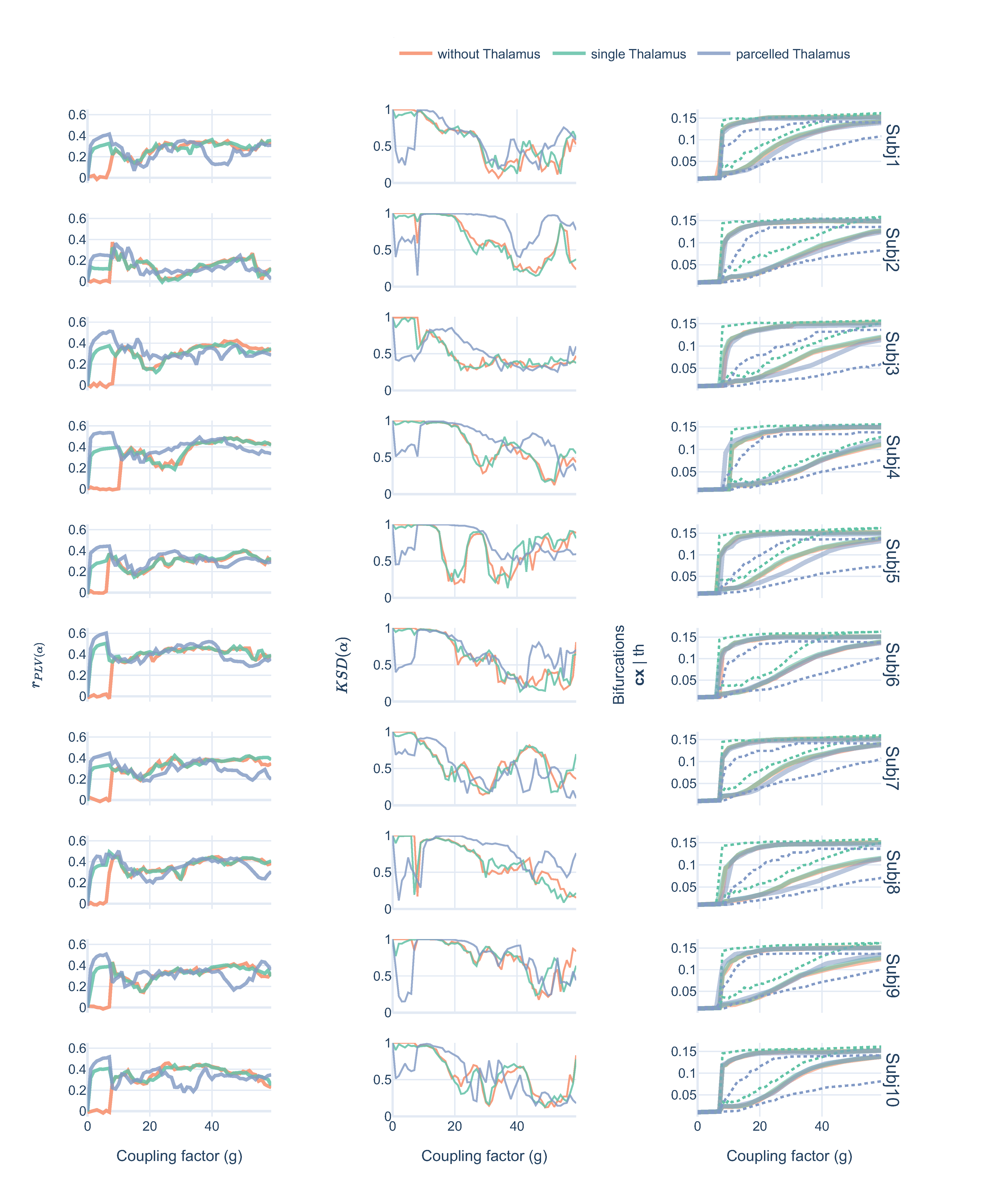

Supplement: S1 Fig — The global behavior in rPLV(first column) was similar for every subject. Note slight differences for subject 2 and subject 8 in which the bifurcation does not match the highest rPLV value. (TIF) [file pcbi.1011007.s001.tif]

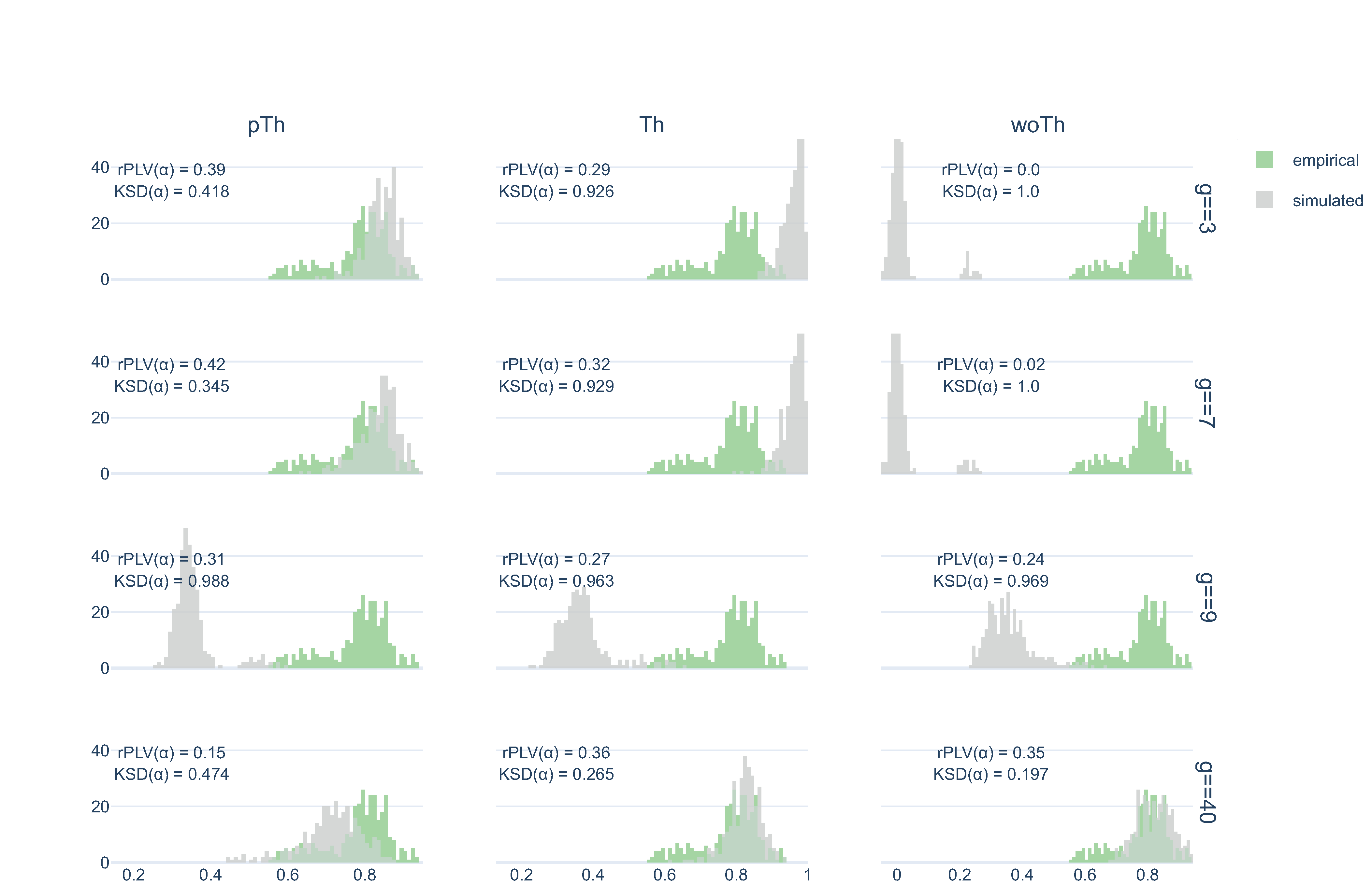

Supplement: S2 Fig — Simulations were performed for subject 1 with high thalamic noise (ηth = 0.022) and in both prebifurcation (g = [3, 7]) and postbifurcation 2 (g = [9, 40]). The three thalamocortical SC (i.e., woTh, Th, pTh) versions were simulated. (TIF) [file pcbi.1011007.s002.tif]

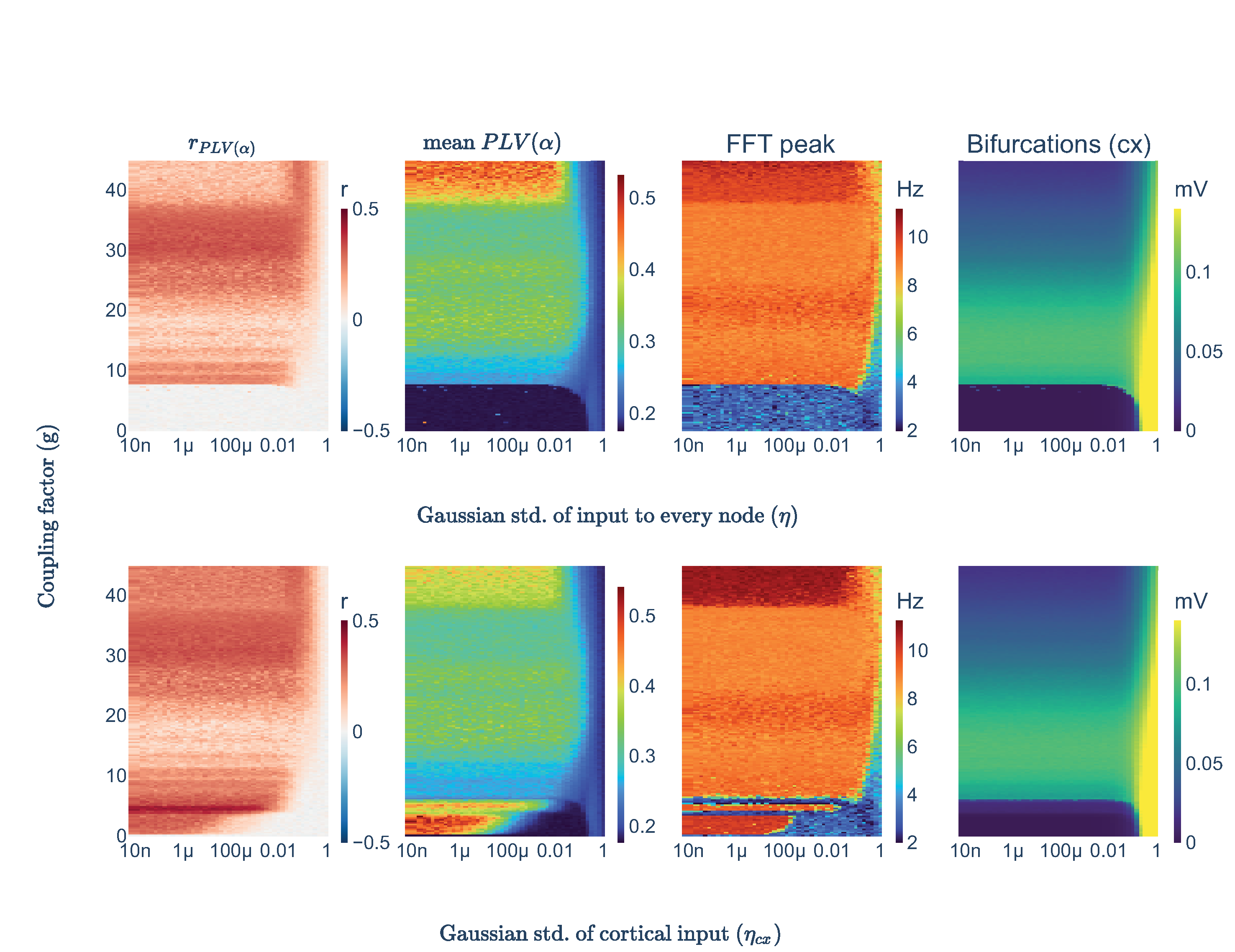

Supplement: S3 Fig — First row, showing simulations where all nodes have the same parametrization (p = 0.09; η = variable). Second row, showing simulations with the thalamus in limit cycle condition pth = 0.15, ηth = 0.09) and a variable noisy input to cortical nodes (pcx = 0.09, ηcx = variable). (TIF) [file pcbi.1011007.s003.tif]

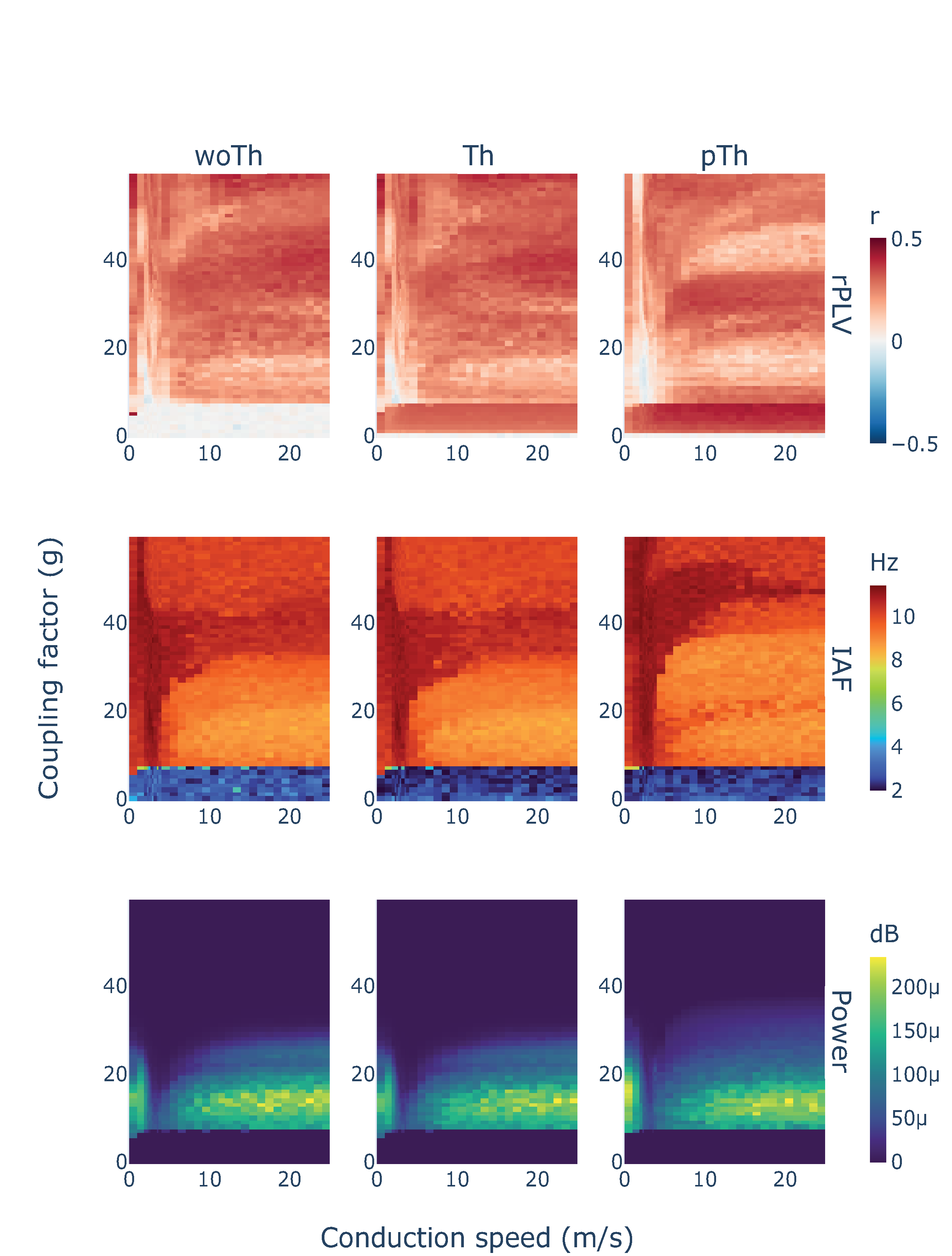

Supplement: S4 Fig — The model was parameterized as in the first in-silico experiment shown in Fig 2F (i.e., the thalamocortical experiment with high thalamic noise pth = 0.09, ηth = 0.022, pcx = 0.09, ηcx = 2.2e-8). Each column shows a set of simulations with a different SC version: woTh, Th, and pTh. The three heatmaps shown per column represent different measures of the same simulation including rPLV(α), IAF as the frequency peak of the averaged spectrum from all nodes, and the power at the frequency peak of the averaged spectrum. (TIF) [file pcbi.1011007.s004.tif]

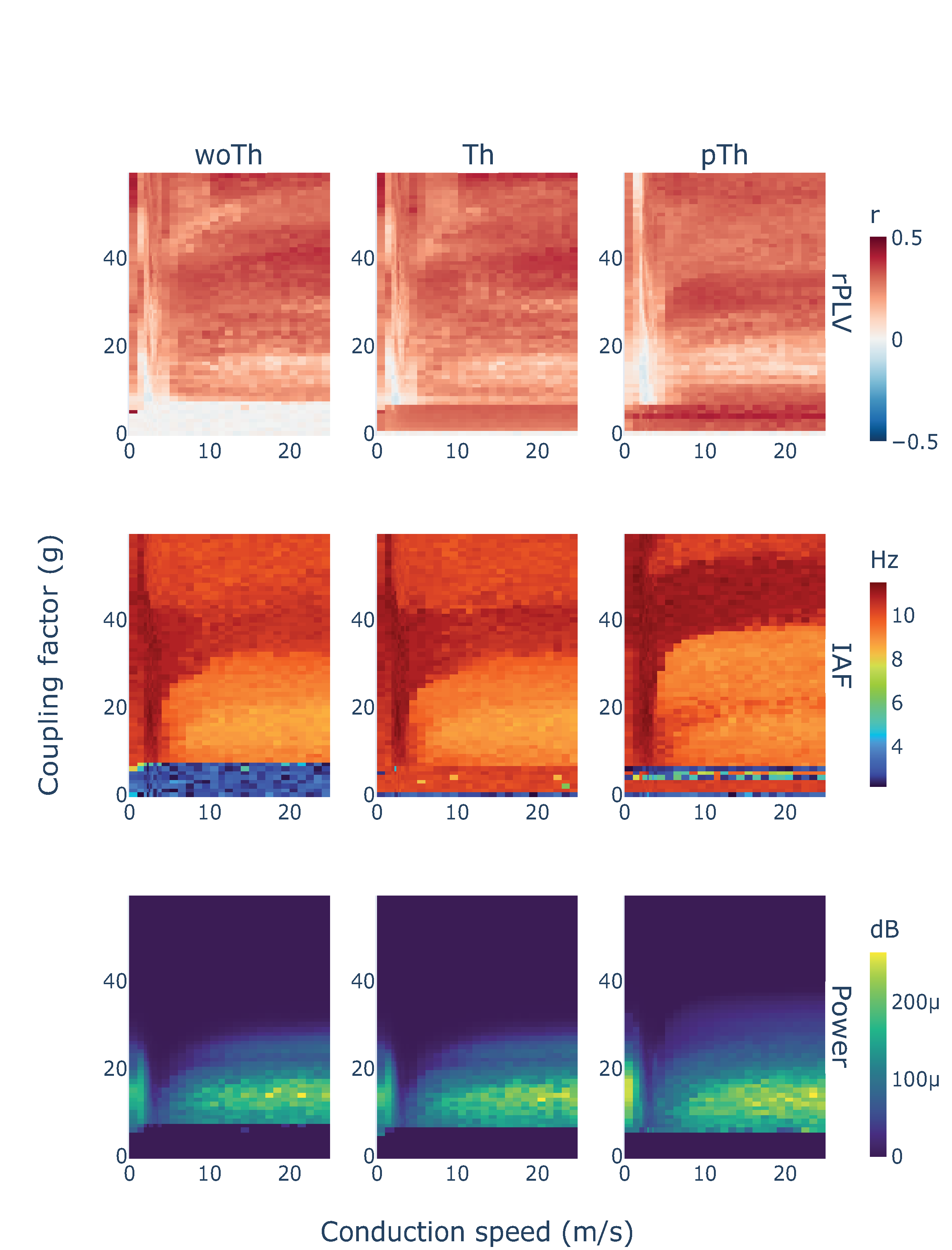

Supplement: S5 Fig — The model was parameterized following the last in-silico experiments to obtain alpha in prebifurcation as shown in Fig 7 second last column (i.e., pth = 0.15, ηth = 0.09, pcx = 0.09, ηcx = 2.2e-8). Each column shows a set of simulations with a different SC version: woTh, Th, and pTh. The three heatmaps shown per column represent different measures of the same simulation including rPLV(α), IAF as the frequency peak of the averaged spectrum from all nodes, and the power at the frequency peak of the averaged spectrum. (TIF) [file pcbi.1011007.s005.tif]
