# Supplementary material for: Modeling the role of the thalamus in resting-state functional connectivity: Nature or structure
Source: PLoS Comput Biol. 2023 Aug 3;19(8):e1011007. doi: 10.1371/journal.pcbi.1011007 (PMC10426958; doi:10.1371/journal.pcbi.1011007)
Supplement: Attachment — Submitted filename: Response to Reviewers @ 03-06-2023.docx [file pcbi.1011007.s007.docx]

Dear reviewers,

Thank you very much for your time and for your interesting and insightful comments on our manuscript titled “Modeling the role of the thalamus in resting-state functional connectivity: nature or structure”. We believe that your comments contributed greatly to enhance the quality and accuracy of our work, and also to strengthen our future research. In general terms, we have discussed your comments, and we followed them to modify the text and figures of the manuscript enhancing its readability and completeness.

**Reviewer #1:** *The authors construct whole-brain network models based on the JR neuronal mass, in order to study the impact of the thalamus. Even though results do not support the initial hypothesis about the importance of the thalamus, the authors still perform a thorough mechanistic exploration of the model from dynamical point of view. As a such the manuscript could be of interest to the field of computational neuroscience that studies the brain as a system, but a more critical analysis of the reasons for the observed shortcomings is necessary.*

*By the construction of the brain network model, all the nodes are equal. Hence* ***the statement that any node could play the role of the thalamus is misleading, unless it is stated that this is the case only for the way the brain model has been constructed.*** *If the thalamus was generated from a different neuronal mass to reflect its distinctive properties, this wouldn’t have been the case. Moreover, if the paths from the thalamus were directional as it is the case in the reality, this also wouldn’t have been the case.* ***The authors should hence refer to the attempts to model specific brain regions as separate entities in the brain network model****, through multi-scale or co-simulation models, such as for example the works using TVB by Meier et al, or the works on the whole brain scaffold modeling by the lab of E. D’Angelo.*

The reviewer is right, in the case of implementing the thalamus with a specific NMM, a spiking neural network (i.e., a multiscale simulation approach), or even implementing directed SC pathways, the results of including or excluding the thalamus could have been different. However, we wanted to keep the modeling as simple as possible to avoid confounding variables that could shadow the impact of thalamo-cortical SC (i.e., structure) and its natural role as relay station in the results of the simulations.

Nevertheless, we will clarify the mentioned statement from discussion (line 259):

“This would mean that in our model, where all neural masses are functionally equal, any node could theoretically play the role of the thalamus.”

And we will introduce a brief paragraph about the possibility of modeling the thalamus differently from other nodes (lines 309-313):

“Additionally, other studies are paving the way towards multiscale computational models in which subcortical areas are implemented with further cellular level details (Meier et al. 2022, D’Angelo & Jirsa 2022, Betzel & Basset 2017, Shaheen et al. 2022). These approaches could be a useful path to extend our knowledge regarding the potential role of the thalamus (and other activating brain regions) in whole brain simulations. “

*Similarly in the line 128 authors say that* ***the thalamus did not show an impact, when in fact it did and it made the fit of the model worse****. The authors should discuss why this could be the case, along the above lines, not to avoid the issue.*

We wrote this statement thinking on the results of the statistical tests regarding postbifurcation range in which only the variable “noise” showed a weak effect [F(1, 9)=5.89, η^2^g=0.006, eps=0.55, p=0.038] compared to the effects found in prebifurcation, for instance the variable noise showed [F(1, 9)=178.25, η^2^g =0.87, eps=1, p<0.0001]. Using a significance level of 0.01, we could deliver that claim.

However, the reviewer’s comment could be oriented towards Figure 1, in which the blue line (i.e., parceled thalamus) showed worse r_PLV_ and KSD values in part of the postbifurcation range, and where the box plots showed also slightly worse performance in both measures for parceled thalamus. These slight differences have a more complex analysis, and we will support our response on the results shown in supplementary figure 2. There, we showed the distributions of correlations in the dFC matrices in different situations, and we observed that two different types of simulations could generate high values of KSD (i.e., poor performance in dFC). On one hand we observed quasistatic simulations in prebifurcation with thalamus as single node (simulations generated a distribution centered around r~1), and on the other hand, we observed randomly changing FC matrices in woTh condition also in prebifurcation (simulations generated a distribution centered around r~0).

Just after the bifurcation (g==9, Fig S2 third row) the dFC distribution for the three thalamic SC implementations resembles the case of woTh in prebifurcation (i.e., low values of FC correlations). In this situation (g==9), all nodes in the network have switched their behaviour into the limit cycle, oscillating autonomously. We believe that the incoming inputs from neighbours become relatively weak in comparison to the autonomous oscillation, and therefore, higher coupling values are needed to establish reciprocal influences that make more consistent the FC matrices (see Fig S2 fourth row).

Based on these observations, we can build our answer to the reviewer´s comment: we believe that having more nodes may complicate establishing those reciprocal influences because there are more autonomous oscillators in the system trying to impose their own dynamics. Therefore, for pTh SC, higher coupling values would be required to establish those significant interactions than for woTh and Th, and it may explain the slight worse performance observed in the boxplots.

We will add a brief statement regarding this analysis at the end the paragraph (lines 136-139):

“In postbifurcation range (in which nodes autonomously oscillate) the values for rPLV were also high, however, the thalamus did not show a significant impact. The slight differences observed in the boxplots may be related to a higher number of autonomous oscillators trying to impose their own dynamics that may make more difficult establishing stable functional interactions between the nodes.”

*A second issue is the importance of* ***time-delays for spectral activation patterns and synchronization*** *as shown by several papers from the lab of V. Jirsa (e.g., the normalization of the connectome).* ***Is JR model sensitive on delays?*** *It should be at least discussed that for oscillatory activity time-delays can be of equal importance as the weights. Within the same line, the importance of the graph theory metrics (and non-identifiability of the regions) appears only because of the simplicity of the model that doesn’t distinguish different brain regions, nor the directed paths from the subcortical regions, and moreover, because the model is either not sensitive to time-delays, or their impact is negligible due to the too high conduction velocity of 15m/s. Lemarechal et al Brain 2022 used the largest ever empirical cohort (close to 1000 patients) to find mean values of ~3.3 m/s, which is also the value in Petkoski & Jirsa 2022 that yields the most recognizable spectral activation patterns. So, the choice of the conduction velocity needs further justification.*

In the first stages of this project, we followed the approach of exploring the parameter spaces for both the coupling factor and the conduction speed. An example of the initial parameter spaces is shown below in Figure 1. As in the manuscript, these parameter spaces can be analyzed separating prebifurcation and postbifurcation ranges. For the space prebifurcation, the model did not seem to be sensible to changes in the conduction speed looking at any of the measures gathered (i.e., rPLV, IAF or power; see Fig 1). However, for the space postbifurcation, conduction speed affected the simulations and showed, in terms of IAF, higher frequency peaks for slower conductions. Importantly, in terms of FC, the best correlations between empirical and simulated FC were found for speed values higher than the proposed 3.3 m/s. Additionally, we noted that given the interaction between conduction speed and coupling factor, it was possible to reproduce all the dynamics shown in the parameter spaces after fixing the speed, but not the coupling. Following this rationale, we fixed the speed averaging the conduction speed derived from the working points derived from our sample.

**Supplementary Fig 4.** Parameter space explorations for conduction speed and coupling factor in the BNM with initial parameters. The model was parameterized as in the first in-silico experiment shown in figure 2F (i.e., the thalamocortical experiment with high thalamic noise pth=0.09, ηth=0.022, pcx=0.09, ηcx=2.2e-8). Each column shows a set of simulations with a different SC version: woTh, Th, and pTh. The three heatmaps shown per column represent different measures of the same simulation including r_PLV_(α), IAF as the frequency peak of the averaged spectrum from all nodes, and the power at the frequency peak of the averaged spectrum.

Considering the reviewer´s comment, we also wonder whether speed has an impact in the model simulations after the parametrization performed in our results sections. To answer this question, we performed again the simulation with the adjusted parameters (Fig 2). Note the differences in the space prebifurcation in IAF. The conclusions for the impact of conduction speed from the previous figure remain. We will include these figures as supplementary material in the manuscript.

**Supplementary Fig 5.** Parameter space explorations for conduction speed and coupling factor in the BNM with final parameters. The model was parameterized following the last in-silico experiments to obtain alpha in prebifurcation as shown in Fig 7 second last column (i.e., pth=0.15, ηth=0.09, pcx=0.09, ηcx=2.2e-8). Each column shows a set of simulations with a different SC version: woTh, Th, and pTh. The three heatmaps shown per column represent different measures of the same simulation including r_PLV_(α), IAF as the frequency peak of the averaged spectrum from all nodes, and the power at the frequency peak of the averaged spectrum.

The recent study referenced by the reviewer from Lemarechal et al. (2022) was not out when we started the project, and it might be of interest for our future research, thanks! Interestingly, the results of that study were achieved using a modified version of the Jansen-Rit model that is usually implemented as the core of the Dynamic Causal Modeling framework.

We will state more clearly the inclusion of interregional delays through tract lengths by editing the following statement in methods (line 405):

“The inter-regional communication introduces heterogeneity in terms of connection strength w_ji_, and conduction delays d_ji_ (i.e., tract length / conduction speed) between nodes i and j”

*Minor comments:*

*- fig. 1 could be made anatomically adequate.*

We replaced the figure with another anatomically informed. This new figure includes a 3D representation of the thalamus with its functional divisions as mentioned in the text. The relay schemes were depicted on top of a coronal slice of the cortex. We will replace the figure caption:

“Main schemes of thalamocortical interaction. A) Functionally distinct parts of the thalamus including the dorsal thalamus, the reticular nucleus as the main component of the ventral thalamus, and the intralaminar nuclei. B) First order relay scheme in which the subcortical/peripheral afferences are relayed to the cortex generally to layers 3 and 4. C) High order relay scheme in which afferences from a cortical region are relayed to another. Coronal slices were acquired from the big brain project (Amunts et al. 2013) using ebrains platform.

*- while rsfMRI FC is discussed as being predictive for aging, lately metrics based on dFC have been shown to have higher predictive value, see e.g. Battaglia et al Neuroimage 2020 and Petkoski et al Cereb Cortex 2023. Also adding some context in why dFC is expected to contain more information would be useful.*

The reviewer is right, some contextualization for the dFC should have been given in the introduction. We will include a set of considerations regarding dFC in the paragraph for FC (lines 40-48):

“Since then, resting-state FC (rsFC) has been used to characterize brain function in health and disease (Spinosa 2022, Hausman 2020, Tang 2022) usually considering it as a static measure. More recently, this approach has been extended to capture the temporal richness of the activity patterns in resting-state through the concept of dynamical FC (dFC; Hutchison 2013, Preti 2017) which has been suggested to reflect ongoing cognitive processing and that may be more informative of brain function and dysfunction (Sadaghiani 2015, Gonzalez-Castillo 2015, Cohen 2018, Petkoski 2023). Both metrics support the characterization of healthy aging, for which a general decrease in static FC, complemented by a slowing and less complex dFC has been shown and related to changes in cognitive performance (Fjell 2015, Betzel 2014, Sala-Llonch 2015, Battaglia 2020). “

*- discarding the tracts longer than 180mm seems way too restrictive unless the authors refer to the Euclidian distance. But I see no reason why to use Euclidian distance instead the actual tract lengths, which could go above 250 mm.*

We decided to restrict the maximum length of the tracts to 180mm to mitigate the impact of the moderate quality of the dw-MRI and to improve the robustness of the tracking algorithm. However, to further test this limitation, we have computed a comparison of the tracking algorithm results for three different values of maximum tract length (180, 250 and 300 mm). Results showed statistical differences between tract lengths of the groups with means equal to 89.56, 95.23, 96.56 mm respectively [F(2, 8316)=24.93, η^2^=0.00596, p=1.6e-11], but no significant differences emerged for the number of streamlines per connection with means equal to 1176.83, 1192.71, 1194.12 streamlines respectively [F(2, 21417)=0.0387, η^2^=0.000004, p=0.96] (see Fig 3).

Additionally, we performed a tractography with the parameters of minimum length of 180 mm and maximum length 300 mm, to obtain a measure of the number of streamlines discarded in the process. We obtained 13678 streamlines in contrast to the 2575616 streamlines found with our protocol 15-180 mm. This makes a 0.5% of streamlines discarded.

Therefore, we believe that preventing the tracking algorithm from false positives at the cost of leaving out from the analysis less than 1% of the streamlines could be a reasonable tradeoff.

**Fig 3.** Violin plots showing the results of tractography with different maximum tract length parameter. Left, results for the number of streamlines. Right, results for the average tract length. Horizontal dashed line indicating the group mean.

*- not clear for which frequency band are the shown PLV values.*

PLV and dFC values are shown for the alpha band. This was stated at the beginning of the results section (line 102) and in the methods section (simulations; lines 410-411). However, the explanation for the computation of FC may result misleading as we stated that FC was computed for all the frequency bands (lines 337-344). This statement was correct, however we restricted eventually our analysis to the alpha band for computational simplicity and being aware that it dominates MEG recordings as explained thereafter in lines 410-411.

To avoid potential confusions, we will add the subscript α where appropriate (i.e., PLV_α_ and KSD_α_), and we will rewrite the FC section in Methods (lines 359-367):

“Source-space signals were then filtered in the alpha band (8-12 Hz) to calculate FC between the time series using the Phase Locking Value (PLV; Lachaux (1999)), and the resulting matrices were averaged into the AAL2 parcellation scheme (Rolls 2015). We restricted the analysis to 1) cortical regions, to avoid the limitations of MEG recordings regarding deep brain signals [104]; and 2) the alpha band, for computational simplicity and being aware that it dominates MEG resting-state recordings.”

*- why the choice of 5s for the windows of dFC? is this robust? and why even a fixed length window for every band? The authors should at least try to justify their choices.*

As we were working with MEG data, we supported our decision in the publication of Liuzzi et al. (2019) in which they used simulated neural activity as ground truths to evaluate the capability of the sliding window approaches with different window lengths and FC metrics (i.e., AEC, PLV, PLI, COH, and iCO) to capture simulated dynamical changes in FC. They found that these metrics worked well for states with moderate to long durations, and especially using longer window lengths. In their discussion they recommended the use of window lengths of at least 3 to 4 seconds to estimate fluctuations in rsFC.

We will include a reference to this article in the Methods section (line 369):

“In addition, we computed dynamical functional connectivity matrices by extracting PLVα on consecutive intervals of 4 seconds of length with the sliding window approach and 50% of overlapping (Liuzzi, 2019), and evaluating the correlation between these PLVα matrices.“

**Reviewer #2**: *In their article, Cabrera-Alvarez et al. simulate a set of coupled Jansen-Rit models as a whole-brain network model in order to compare the model functional connectivity with empirical functional connectivity obtained with MEG data. They use structural connectivity obtained using diffusion MRI to couple the local regional models and scaling parameter g which is a classical approach in these types of models. Then they compare the model-empirical FC fit between three different versions of the local connectivity in the thalamus - namely without, with a single node and with a parcellated node thalamus - for values of g that correspond to pre- (when the models behave as damped oscillators) and post - (in limit cycle oscillators regime) bifurcation space. They also compare model-empirical FC for each case with low and high values of noise input to the thalamic node(s) that is supposed to represent afferences from the reticular system and argue that best fits are obtained in the pre-bifurcation region with high noise input to parcellated thalamus. They then use this result to claim that thalamus could be playing a driving role in the emergence of overall RS-FC.*

*I find that the simulations in the paper are performed with adequate detail and correctly. However, I found the rationale/motivation for the type of modelling/analysis performed lacking. I also found the interpretation of the results problematic especially with regards to the final conclusion and the title of the paper.*

*My first major concern is with* ***the emphasis the authors put on the pre-bifurcation*** *region. Looking at their results in this region, the model-empirical FC fit for parcellated thalamus case with high noise is significantly higher than without thalamus & with single node thalamus models. However, the r_PLV_ values in the post-bifurcation region are in the same range as these highest pre-bifurcation values (pTh + high noise). Therefore, it’s not clear why the authors focused on the pre-bifurcation region specifically later on, i.e.,* ***what additional biological/empirical evidence*** *(power spectrum for instance?)* ***implies that the pre- and not post-bifurcation region is the optimal one*** *is not clear.*

The reviewer is right, the activity of the Jansen-Rit model in postbifircation range has been often associated to a state of generalized epileptic seizure in the computational neuroscience literature (Wedling et al. 2016, Breakspear et al. 2006). Indeed, the power spectra in Figure 4B, where the second and fourth columns represent postbifurcation simulations, show excessively synchronized intra-node oscillatory behaviour in alpha band with respect to empirical recordings.

We will add these ideas to our discussion section (lines 250-254):

“More importantly, the postbifurcation range has been often associated to a state of generalized epileptic seizure (Wendling et al., 2016, Breakspear et al., 2005} due to its highly synchronized intra-node oscillatory activity (see the spectra at postbifurcation in Fig 4B) that is not the empirical target of this study (i.e., MEG resting state). Taken together, these ideas led us to focus on the prebifurcation range.”

Additionally, other computational studies have proposed that the brain might work at criticality regimes between oscillatory states, and this implies that part of the simulated regions are operating at prebifurcation (Deco et al. 2017). We discussed this idea in our last section relating their proposed criticality behaviour with the driving thalamocortical mechanisms implemented in our model. This is another idea that led us to focus in prebifurcation.

*Instead of using global FC metrics,* ***authors could consider specific FCs*** *and see how they match between the model & empirical data. For instance, which model - parcellated or single-node thalamus - gives a better matching to empirical* ***thalamocortical FC*** *could be explored to fine-tune both the parameter-space (g) as well as the choice of pre-vs-post bifurcation regime.*

Two main reasons led us to consider global FC metrics. First, we are working with MEG and the reconstruction of deep brain signals is not recommended due to the attenuation of the signal as a function of distance, the deep structures being closed fields generators, and deep activity being overshadowed by superficial neocortical generators (Bénar et al. 2021). Therefore, the regions included in the empirical functional connectivity matrices and in the comparisons with simulated FC were limited to the cortical ones of AAL atlas.

We have reorganized the methods section to state clearly the use of only cortical regions in the FC analysis moving this information from the Simulations subsection of Methods (lines 439-442) to the Functional Connectivity subsection (lines 363-365):

“We restricted the analysis to 1) cortical regions, to avoid the limitations of MEG recordings regarding deep brain signals (Bénar et al. 2021); and 2) the alpha band, for computational simplicity and being aware that it dominates MEG resting-state recordings.” ­

On the other hand, our hypothesis regards the impact of thalamus on cortical FC. We were considering the potential role of thalamus as a dynamical relaying node that indirectly fosters the synchronization between cortical regions. We were not so interested in the specific relationship that cortical regions stablish with the thalamus, and therefore, we aimed at the global FC between cortical regions from the beginning.

*Authors show that instead of the thalamus, using a parcellated cerebellum gives the same levels of model-empirical FC fits as with the thalamus. They use this result to say that the specific structure in the thalamus does not matter and it’s the noise. But the flip side of this observation is also that, within the limited confines of the metrics (FC and dFC similarity) used by the authors and the model,* ***thalamus is not unique****. So* ***how could one make a claim****, based purely on these observations,* ***that the thalamus is performing a driving role in emergence of FC? Perhaps it’s the cerebellum?*** *Either the authors should moderate their claims about the role of the thalamus in the article or they should use additional metrics (perhaps local FC instead of global, brain-wide FC?) to make a distinction between the thalamus vs cerebellum comparisons.*

The key to understand the rationale behind this claim is that we know from theory that it would be biologically plausible to model the thalamus with higher noisy inputs due to its sensory relays and the participation in RAS system. However, to our knowledge, there is no other reason to implement higher noisy inputs into the cerebellum.

Therefore, the control experiment in which we implement high noise into cerebellar regions was a priori biologically incorrect, however, it gave us the opportunity to test independently the effects of structure (comparing the thalamo-cortical SC to the cortico-cerebellar SC) separating it from the effects of noise that was kept fixed. This way and observing that the cerebellum with the same levels of noise could generate similar effects on FC than thalamus, we derived that the specific structure of thalamus was not the key to explain the changes in FC shown in the results.

*Also, I was curious to see* ***what happens if both the cerebellum and thalamus are parcellated*** *(that would be closer to biological reality anyway)?*

We used the parcellated structures as the default ones (i.e., the ones included in the AAL3 atlas). Therefore, woTh, Th and pTh include the cerebellum parcellated; and woCer, Cer and pCer include the thalamus parcellated. Consequently, the models “pCer” and “pTh” include both the thalamus and cerebellum parcellated. This was stated in the Structural connectivity subsection of Methods (see Figure 8). However, we will try to clarify this aspect in the methods section (line 388):

“These three versions of the structural connectomes [woTh, Th, and pTh] are represented in figure 8 and included the cerebellum parcelled into its nuclei.”

And in the Results section (lines 147-148):

“We built three SC versions per subject: parceled cerebellum (pCer), single node cerebellum (Cer) and without cerebellum (woCer), all of them including the thalamus parcellated.”

*It is not clear how the authors* ***chose the noise levels****. There should be an explanation for the values chosen by the authors.*

Two noise levels were implemented in the model. The initial high noise level for the thalamus (η_th_=0.022) was selected following David & Friston (2003), while the background noise (η=2.2e^-8) was selected to avoid zero derivatives in fixed state of the JR model and maintaining a significant difference to the thalamic input.

We will add the following sentence to the Simulation subsection in Methods (lines 431-433) to clarify this:

“The higher noise level was determined following previous research (David & Friston, 2003), while the lower was chosen to avoid flat signals in the fixed state of the JR model.”

*The* ***readability of the manuscript*** *can be improved is significantly overall.* ***Sub-figures can be labelled A, B*** *etc.* ***Figure captions*** *at the moment are extremely inadequate and they can’t be understood without going through the results section. That should not be the case.*

Thanks for the comment. We updated several graphs introducing labels for subfigures, and we rewrote the captions for a better understanding.

*I take it that the horizontal blue lines in the FFT plots in figures 5 and 6 represent bifurcation thresholds only? I would suggest to use a different color that’s not included in the colormaps of these figures for the lines in that case.*

The reviewer is right, the horizontal blue lines in figures 5-6 represent bifurcation thresholds. However, they are part of the heatmaps resulting from simulations, they were not added ad-hoc.
